# Postpartum readmission for maternal infection or neonatal adverse events in women randomised to outpatient balloon versus inpatient prostaglandin for induction of labour: a post-trial follow up study

**DOI:** 10.1186/s12884-026-09084-3

**Published:** 2026-04-20

**Authors:** Sara Carlhäll, Jane Alsweiler, Malcolm Battin, Jessica Wilson, Lynn Sadler, John Thompson, Michelle Wise

**Affiliations:** 1 Department of Obstetrics, Gynaecology and Reproductive Sciences, Faculty of Medical and Health Sciences, University of Auckland, Auckland, New Zealand; 2 Department of Paediatrics, Child and Youth Health, Faculty of Medical and Health Sciences, University of Auckland, Auckland, New Zealand; 3 Women’s Health, Te Whatu Ora Te Toka Tumai, Auckland, New Zealand; 4 Department of Biomedical and Clinical Sciences, Linköping University, Linköping, Sweden; 5 Clinical Department of Obstetrics and Gynaecology in Linköping, Region Östergötland, Linköping, Sweden

**Keywords:** Controlled clinical trial, Labour, Induced, Delivery, Obstetric, Postpartum infection, Infant, Newborn, Patient readmission

## Abstract

**Background:**

Induction of labour is common, and much has been studied about different methods and clinical outcomes for mothers and babies. However, many trials follow outcomes only until hospital discharge. The recently published OBLIGE multicentre randomised controlled trial of 1087 participants found no differences in adverse events for mothers or their babies in groups randomised at term to outpatient balloon catheter versus inpatient vaginal prostaglandin E2. The aim of this analysis was to evaluate maternal and neonatal readmissions from hospital discharge to six weeks after birth.

**Methods:**

A post-trial follow up of the OBLIGE trial. The clinical records of a subset of participants for whom notes were accessible were reviewed (564 mothers and 477 babies). The primary outcome was maternal readmission to hospital up to six weeks after birth (including a hospital visit of 3 h or more) for infection requiring antibiotics. The secondary outcome was neonatal readmission to hospital up to six weeks after birth (including a hospital visit of 3 h or more) for any reason.

**Results:**

Of women randomised to outpatient balloon, 6.3% were readmitted with an infection compared to 6.5% in the inpatient prostaglandin group (*p* = 0.99). Of babies born to mothers randomised to outpatient balloon, 10.2% were readmitted compared to 11.7% in the inpatient prostaglandin group (*p* = 0.59).

**Conclusions:**

In this subset of participants in the OBLIGE trial, there were no differences in maternal readmission for infection, or neonatal admission for any reason, up to six weeks after birth. Consideration of readmission risk for mothers and babies after outpatient balloon induction of labour does not seem to be a reason not to routinely offer outpatient balloon induction of labour.

**Trial registration:**

The OBLIGE trial was registered with the Australia New Zealand Clinical Trials Registry (ANZCTR) on 06/06/2016 (ACTRN: 12,616,000,739,415). https://www.anzctr.org.au/Trial/Registration/TrialReview.aspx?id=370330&isReview=true

## Background

During the postpartum period, defined as the first six weeks after childbirth, women are particularly susceptible to infection [1]. Postpartum infection has been reported to affect 1.1% to 7.5% of all women [2–5]. However, the global incidence is difficult to assess, due to different definitions and obstetric management, and the epidemiology of maternal postpartum infections is poorly understood [2, 3]. Infection is an important preventable cause of maternal morbidity and mortality. Still, sepsis during pregnancy and puerperium is one of the leading causes of maternal morbidity and mortality worldwide [6]. Therefore, it is of great importance to assess whether new interventions increase risk of postpartum infections. Induction of labour (IOL) has been described as a risk factor for postpartum infections [7].

In contemporary obstetrics, the increasing number of women with induced labour has an impact on limited resources at birthing suites and outpatient IOL has been introduced as an alternative setting. To assure the safety of outpatient IOL compared to inpatient IOL, randomised controlled trials (RCT) have been performed [8]. An individual participant data meta-analysis of RCTs comparing balloon catheters versus vaginal prostaglandins concluded that few trials include adverse long-term maternal outcomes, such as infection, nor long-term neonatal outcomes, and encouraged further investigation [9].

The “Outpatient Balloon versus Inpatient Prostaglandin for Induction of Labour” (OBLIGE) trial, was a multicenter RCT in New Zealand, comparing outpatient balloon catheter with inpatient prostaglandin (PG) E2 [10]. In the OBLIGE trial outcomes were assessed until discharge from hospital after birth. A post-trial follow up, defined as extended follow-up which starts at the end of the scheduled period of the original trial, is desirable and may add scientific value to the evaluation of healthcare interventions [11].

In this post-trial follow up of the OBLIGE trial, we aimed to evaluate maternal readmission for infection, and neonatal readmission for any reason, within six weeks after birth, in women randomised to outpatient balloon catheter or inpatient PG for IOL at term. We hypothesized that there was no difference in readmissions between the groups.

## Methods

This is a post-trial follow up of the OBLIGE trial, a multi-centre, superiority, RCT in 11 public hospitals in New Zealand, covering 50% of annual births nationally. Pregnant women with planned IOL at term (≥ 37 + 0 gestational weeks) were randomised to start IOL either with outpatient balloon catheter or with inpatient PG E2. The OBLIGE study population included 1087 pregnant women induced between October 2017 and November 2021, with intact membranes and a non-urgent maternal or fetal indication for IOL, where induction was elective or planned more than 48 h in advance and outpatient care was not considered contraindicated. Informed written consent was obtained from all participants. Further inclusion criteria were: singleton pregnancy, vertex presentation, normal cardiotocography/non stress test, Bishop score < 7; able to remain within one hour of hospital with someone who could speak sufficient English to communicate with hospital staff. Women with previous cesarean birth and pregnancies with major fetal anomaly or suspected severe fetal growth restriction were excluded. Randomization was stratified by research centre and parity [10].

The primary outcome was maternal readmission or visit to hospital lasting three  hours or more, within six weeks after birth, with any infection treated with antibiotics. The secondary outcome was neonatal readmission or visit to hospital lasting  three hours or more, within six weeks after birth, for any reason.

Maternal and neonatal demographic and clinical data included in OBLIGE are detailed in the original publication [10]. For the current study, new labour time estimates were calculated based on data included in the original OBLIGE trial and additional maternal and neonatal data were collected by electronic medical record review. This was performed for a subset of 564 mothers who gave birth at Te Toka Tumai Auckland Hospital and two hospitals in Waitematā region, and a further subset of 477 babies born at Te Toka Tumai Auckland Hospital, for whom medical records were available to the researchers after the OBLIGE trial was complete. The current study outcomes of maternal and neonatal readmissions after six weeks were not included in the Trial Registry and required an ethics amendment to collect these additional data.

The mothers were identified by merging the women´s national health index number to any admission of a stay for at least three hours in any of these three hospitals within six weeks after birth. A medical record review was done to obtain data on the number of and causes of readmission. Some women had several different readmission visits and/or diagnoses at each readmission visit. Women who were readmitted several times were counted only once. Each participant could appear under more than one reason for each readmission but only once under each reason. The causes for readmission were categorized into any postpartum infection or non-infection cause (such as hypertension or breastfeeding problems). For the purpose of this study, post-partum infection was defined as clinical signs of infection and prescribed oral and/or intravenous antibiotics by the clinician assessing the woman. Infections were classified as endometritis, wound (caesarean section or perineum), mastitis, urinary tract or other.

The number of women with readmission or hospital visit, with and without infection, as well as type of infection, and if puerperal sepsis was present as coded by ICD-10-CM, were collected. Smoking in early pregnancy, smoking at birth, and breastfeeding at time of discharge from hospital, were also collected as they are considered to be associated with maternal and neonatal infection, and neonatal readmission.

The babies were identified by merging the babies’ national health index number to any admission or a hospital stay at Te Toka Tumai Auckland Hospital for at least three hours within six weeks after birth. The reasons for readmission were classified as jaundice, infection, feeding difficulties, or other (such as unsettled babies with normal findings or trauma). The number and reasons for readmission for the babies were collected. Some babies had several different readmission visits and/or diagnoses at each readmission; babies that were admitted several times were counted only once.

### Statistics

Statistical analysis was performed using SAS v 9.4 (SAS Institute, Cary, NC, USA). The maternal and neonatal baseline and birth characteristics were described by frequencies if categorical or mean and standard deviation if continuous and normally distributed or median and interquartile range if not normally distributed. The participants were analysed according to the assigned intervention group at randomization. In this study, 30/287 (10%) of participants allocated to the outpatient balloon and 11/277 (4%) allocated to inpatient prostaglandins did not receive their randomised allocation.

The incidence of maternal and neonatal readmissions and causes were calculated for each randomised group. Analysis was performed using Chi-square to test for differences between groups for categorical variables. For continuous variables, comparisons were performed using a t-test for normally distributed variables and a Wilcoxon rank-sum test for those with a non-normal distribution. A p value of 0.05 was considered statistically significant.

### Ethics

The OBLIGE trial received Health and Disability Ethics Committee New Zealand ethical approval on November 23, 2016 (16/CEN/121) and the current study received approved ethical amendment on December 15, 2022 (16/CEN/121). This research was conducted in accordance with the ethical principles outlined in the Declaration of Helsinki.

## Results

**Fig. 1:**
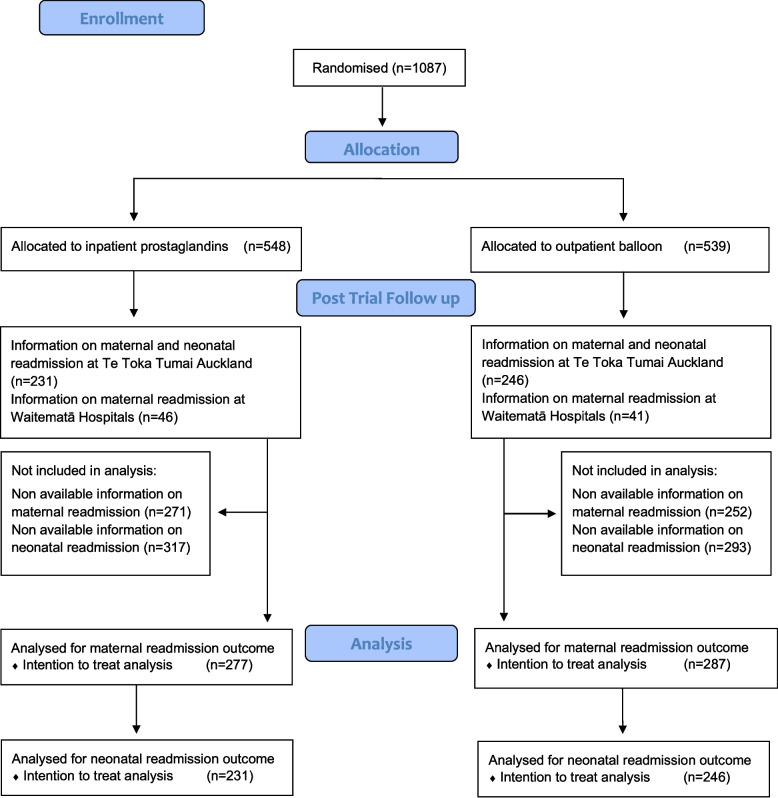
Flow diagram for post trial follow up of the OBLIGE trial

The flow of participants is depicted in Fig. 1.

The maternal and neonatal characteristics are presented in Table 1. In the current study population of 564 women, 277 were randomised to the inpatient PG group and 287 to the outpatient balloon group. Out of the 477 babies, 231 were born to mothers randomised to inpatient PG and 246 to mothers randomised to outpatient balloon. There were no clinically relevant differences in the maternal or neonatal characteristics between the groups except for ethnicity; there was a greater proportion of women of Māori and Asian ethnicity and fewer women with European ethnicity in the outpatient balloon group compared to the inpatient PG group. Maternal and neonatal characteristics were similar to the study population in total OBLIGE cohort [10].

**Table 1:** Participant characteristics by randomised induction method

	**Inpatient prostaglandin** **(*N* = 277)**		**Outpatient balloon** **(*N* = 287)**		
**Maternal and neonatal characteristics**	**n or mean**	**% or SD**	**n or mean**	**% or SD**	***P*-value**
Age, (years)	33	4.8	33	4.9	0.78
Age, categories (years)					0.63
< 35	180	65%	192	67%	
≥ 35	97	35%	95	33%	
BMI(kg/m^2^)	26	6.2	27	6.7	0.36
BMI, categories (kg/m^2^)					0.42
< 25	147	53%	143	50%	
25–29	68	25%	66	23%	
≥ 30	62	22%	78	27%	
Ethnicity					0.02
Māori	12	4.3%	28	9.8%	
Pacific	25	9.0%	23	8.0%	
Indian	33	12%	32	11%	
Asian	30	11%	50	17%	
European	167	60%	145	51%	
Other	10	3.6%	9	3.1%	
New Zealand Index of Multiple Deprivation					0.87
1–2 (least deprived)	67	24%	62	22%	
3–4	73	26%	71	25%	
5–6	67	24%	71	25%	
7–8	47	17%	55	19%	
9–10 (most deprived)	23	8.3%	28	9.8%	
Nulliparous	221	80%	231	81%	0.83
Gestational age at start of IOL (weeks)	40	1.2	40	1.3	0.15
Gestational age at start of IOL categories (weeks)					0.56
37 + 0 to 38 + 6	62	22%	75	26%	
39 + 0 to 40 + 6	138	50%	133	46%	
≥ 41 + 0	77	28%	79	28%	
Indication for IOL					0.64
Post date	80	29%	78	27%	
Diabetes mellitus	80	29%	91	32%	
Small for date or IUGR	26	9.4%	25	8.7%	
Hypertension	19	6.9%	18	6.3%	
Late maternal age	17	6.1%	23	8.0%	
Large for date/macrosomia	11	4.0%	7	2.4%	
Reduced fetal movements	19	6.9%	16	5.6%	
In vitro fertilization pregnancy	6	2.2%	2	0.7%	
Other^a^	19	6.9%	27	9.4%	
Bishop score at start of IOL					0.55
0—2	114	41%	125	44%	
3—4	125	45%	117	41%	
5—6	37	13%	44	15%	
Lead maternity carer type					0.45
Independent Midwife	169	61%	176	61%	
Hospital Midwife	58	21%	69	24%	
Private Obstetrician	50	18%	42	15%	
Hospital					0.19
Te Toka Tumai Auckland	231	83%	246	86%	
North Shore	44	16%	35	12%	
Waitakere	2	0.7%	6	2.1%	
Smoking at booking	7	2.5%	11	3.8%	0.39
Smoking at birth	6	2.2%	7	2.4%	0.84
Breastfeeding at time of discharge					0.17
Exclusively breast fed	215	78%	211	74%	
Partial	54	20%	70	24%	
Missing	8	2.9%	6	2.1%	
Birthweight (grams)^b^	3481	432	3525	500	0.30
Neonatal Sex^b^					0.12
Female	114	50%	139	57%	
Male	117	51%	107	44%	

*N*=231 Inpatient prostaglandin

*N*=246 Outpatient balloon

*BMI* Body Mass Index, *IOL* Induction of labour, *IUGR* Intrauterine growth restriction, *SD* Standard deviation

^*a*^Other reasons: Antepartum haemorrhage, Oligohydramnios, Obesity, Obstetric cholestasis, Maternal medical condition, Maternal request, fetal condition, other

^b^Te Toka Tumai Auckland Hospital participants only

Labour and birth characteristics are described in Table 2.

**Table 2:** Maternal and neonatal labour and birth characteristics by randomised induction method

	**Inpatient prostaglandin** **(*N* = 277)**		**Outpatient Balloon** **(*N* = 287)**		
**Maternal and neonatal characteristics**	**n or median**	**% or IQR**	**n or median**	**% or IQR**	***P-*value**
Need for second method of induction	13	4.7%	77	27%	<.0001
Caesarean section	110	40%	116	40%	0.86
Artificial rupture of membranes	180	65%	237	83%	<.0001
Epidural anaesthesia	187	68%	222	77%	0.009
Oxytocin	167	60%	211	74%	0.0008
Chorioamnionitis in labour	10	3.6%	17	5.9%	0.20
Postpartum haemorrhage ^a^	115	42%	134	47%	0.22
Postpartum endometritis ^b^	3	1.1%	4	1.4%	0.74
	*N* = 266		*N* = 273		
Time from start of IOL to birth (hours; median, IQR)	27	(14–36)	36.4	(28–47)	<.0001
	*N* = 274		*N* = 286		
Time from rupture of membranes to birth (hours; median, IQR)	8.3	(2.9–13)	10.8	(6.0–16)	<.0001
	*N* = 247		*N* = 260		
Time in active labour (hours; median, IQR)	5.6	(2.9–8.7)	6.3	(3.0–8.6)	0.28
Time from birth to maternal discharge (hours; median, IQR)	34	(5.1–54)	40.5	(13–68)	0.004
Total time in hospital for mother (hours; median, IQR)	59	(35–97)	64	(38–102)	0.30
Admission to NICU^c^	4	1.7%	12	4.9%	0.06
*if yes, length of stay, (hours; median, IQR)*^*c*^	55	(30–308)	36.4	(28–53)	
Admission to NICU > 4 h^c^	4	1.7%	12	4.9%	0.06
Need for respiratory support^c^	4	1.7%	7	2.8%	0.42
Apgar < 7 at 5 min ^c^	1	0.4%	6	2.4%	0.07

*N*=231 Inpatient prostaglandin

*N=*246 Outpatient balloon

*IQR *Interquartile range,* NICU *Neonatal intensive care unit

^a^Defined as total estimated blood loss ≥ 500 mL

^b^Diagnosed before discharge from hospital

^c^Te Toka Tumai Auckland Hospital participants only

Similar findings as in the total OBLIGE cohort were seen [10]. In the subset of participants in the current study, there was no difference in caesarean section between the groups, nor was there a difference in neonatal adverse events. The women allocated to outpatient balloon catheter spent longer time from start of IOL to birth (*p* < 0.001), from rupture of membranes to birth (*p* < 0.001) and from birth to discharge (median time 33.9 vs 40.5 hours) (*p* = 0.004) than women allocated to inpatient PG, but no difference in time in active labour was seen.

Maternal postpartum readmissions for the 564 women are described in Table 3.

**Table 3:** Maternal postpartum readmission by randomised induction method

	**Inpatient prostaglandin** **(*N* = 277)**		**Outpatient Balloon** **(*N* = 287)**		
	**n**	**%**	**n**	**%**	***P-*value**
Readmission^a^	30	11%	31	11%	0.99
Readmission for infection^a^	18	6.5%	18	6.3%	0.91
Type of infection^b^					
Endometritis	6	2.2%	5	1.7%	
Wound	7	2.5%	7	2.4%	
Mastitis	6	2.2%	3	1.0%	
Urinary tract	1	0.4%	2	0.7%	
Other	3	1.1%	5	1.7%	
Sepsis	5	1.8%	4	1.4%	
Readmission for non-infectious cause ^c^	14	5.1%	16	5.6%	0.78

^a^Women who were readmitted several times were counted only once

^b^Types of infection are not mutually exclusive. Participants may have been admitted for more than one type

^c^Examples of non-infectious causes were hypertension and breastfeeding problems

In both groups 11% were readmitted. Of women randomised to outpatient balloon, 6.3% were readmitted with an infection and need for antibiotics compared to 6.5% of women randomised to inpatient PG (*p* = 0.91).

Neonatal readmissions for the 477 infants are described in Table 4.

**Table 4:** Neonatal postpartum readmission^d^ by randomised induction method

	**Inpatient prostaglandin** **(*N* = 231)**		**Outpatient Balloon** **(*N* = 246)**		
	**n**	**%**	**n**	**%**	***P*-value**
Readmission^a^	27	12%	25	10%	0.59
Readmission reason^b^					
Infection	9	3.9%	5	2.0%	
Jaundice	7	3.0%	3	1.2%	
Feeding problems	6	2.6%	7	2.8%	
Other^c^	13	5.6%	14	5.7%	

^a^Neonates who were readmitted several times were counted only once

^b^Admission reasons are not mutually exclusive. Participants may have been admitted for more than one reason

^c^Such as: unsettled baby with normal findings or trauma

^d^Te Toka Tumai Auckland Hospital participants only

Of those born to mothers randomised to outpatient balloon, 10% were readmitted, compared to 12% in the babies of the women randomised to inpatient PG (*p* = 0.59).

## Discussion

In this post-trial follow up of the OBLIGE trial we did not find a difference in maternal postpartum readmission for infection, nor neonatal readmission for any reason, between the women randomised to inpatient PG and the women randomised to outpatient balloon catheter IOL.

The OBLIGE trial concluded that the use of balloon catheter in an outpatient setting does not seem to increase the rate of adverse events for mothers or babies compared to inpatient prostaglandins and can be offered routinely [10]. A similar conclusion was drawn in two Australian trials comparing outpatient balloon with inpatient PG for IOL, including 101 and 448 women respectively, but postpartum readmission/adverse events after discharge from hospital was not reported in these trials [12, 13]. When introducing new clinical procedures, it is important to ensure all aspects of the safety of the new routines and to ensure that the safety of the patients is not compromised in the long term. Since the OBLIGE trial ended at discharge from hospital after birth, and given the recommendation that randomised trials on IOL should include long term outcomes [14], we performed a follow up study of a sub-group of mothers and their babies up to six weeks following childbirth.

The postpartum period is a sensitive time, marked by an increased susceptibility to different types of maternal infections [15]. IOL is described as a risk factor for maternal postpartum infection in observational data [7, 16]. Whether this risk is related to a specific induction method or setting has been studied with conflicting results [17, 18]. It has been speculated that mechanical induction methods, in the form of foreign bodies such as balloon catheter, might increase the risk of infection, compared to pharmacological induction methods. A meta-analysis conducted in 2008 by Heinemann et al. of 30 RCTs evaluating maternal infection (4468 participants) and eight RCTs of neonatal infection (1745 participants) found a higher risk of maternal and neonatal infection in women who underwent IOL with mechanical methods compared to pharmacological agents alone [17]. The risk remained increased for maternal, but not neonatal, infection when Foley balloon catheter alone was compared with pharmacological methods [17]. Since this meta-analysis included studies published before 2006, when induction rates were lower and the indications for IOL were different, the results may not be applicable to a contemporary obstetric population.

Similar to our findings, a 2012 meta-analysis of 26 RCTs (5563 participants) by McMaster et al. comparing inpatient Foley balloon catheter with locally applied PG, showed that Foley catheter for IOL was not associated with maternal or neonatal infection [18]. The incidence of pooled maternal infection was 8.8% for Foley balloon and 9.0% for locally applied PG and for neonatal infection the incidence was 3.2% vs 3.6% [18]. A secondary analysis of a RCT reporting on maternal and neonatal composite outcomes in 2376 low risk women having IOL found no difference by method of cervical ripening for maternal outcomes, but Foley catheter, alone or concurrent with PG, was associated with lower risk of neonatal adverse outcomes compared with PG alone [19].

Outpatient vs inpatient settings for IOL have been compared in several RCTs and some have included maternal infection during or close to childbirth as an adverse outcome [20, 21]. In two meta-analyses of RCTs comparing outpatient and inpatient cervical ripening with balloon catheter, the risk of chorioamnionitis or endometritis did not differ between groups. Postpartum infection after hospital discharge was not reported [20, 21]. A recent Cochrane network meta-analysis included one trial (48 participants) which reported on neonatal infection and found no increased risk for neonatal infection nor serious neonatal morbidity in outpatient induction with Foley balloon catheter [22]. However, we have not found any RCTs of outpatient IOL evaluating postpartum readmission for maternal infection or for neonatal adverse events up to  six weeks after birth.

Chorioamnionitis during labour is a risk factor for postpartum infection for both mothers and babies, and associated with adverse long term neonatal outcomes [23–25]. Neither the total OBLIGE cohort nor the present subset of the OBLIGE cohort showed a difference between groups in chorioamnionitis in labour or postpartum endometritis before discharge from hospital [10]. Other interventions and complications during labour that have been associated with increased risk for postpartum infections are caesarean section, instrumental vaginal birth, prolonged rupture of membranes and postpartum hemorrhage [5, 26, 27]. The OBLIGE study as well as the present sub-study found these outcomes did not differ between the randomised groups.

The overall prevalence of postpartum infection in this subset of women with a non-urgent indication for IOL was 6.4%, which is similar to previous studies [2, 5]. The most common maternal infections were endometritis, wound infection and mastitis, also consistent with other studies [3, 4]. In the current study, the prevalence of readmission with sepsis within six weeks after childbirth was low (1.6%) compared to other studies [28].

Although the neonatal readmission rate did not differ between the groups, we found a higher neonatal readmission rate (10.9%) compared to observational studies: one nested case–control study of healthy term infants found a readmission rate of 2.2% [29] and two cohort studies of pre-term babies found a readmission rate of 4.0% [30, 31]. This may have been influenced by our definition of readmission (three hours or more), and differences in the study cohorts and the health care systems.

Limitations of this analysis include a reduced sample size due to having access to readmission data for only half of the original study population in the OBLIGE trial (544 out of 1087). Moreover, due to access to records at only three hospitals, we may have underestimated the prevalence of maternal readmission if patients were readmitted to a hospital other than their birthing hospital, however, this is unlikely given the three hospitals cover the whole of the city of Auckland and each offers both maternity and paediatric admissions. A strength is that the current cohort was representative of the total OBLIGE cohort by demographic and clinical characteristics and can therefore be considered representative.

## Conclusion

In this post-trial follow up of the OBLIGE trial, we found no difference in maternal readmission for infection, and neonatal readmission for any reason, within six weeks after birth, in women randomised to outpatient balloon catheter or inpatient PG for IOL at term. These results support and extend the conclusion from the original trial that outpatient balloon IOL does not increase the risk of adverse events for mothers or babies up to six weeks after birth and can thus be offered routinely.

## Data Availability

The datasets used and/or analysed during the current study are available from the corresponding author on reasonable request.

## Abbreviations

IOL: Induction of labour
RCT: Randomised controlled trial
PG: Prostaglandin
